# The relationship between cognitive function and muscle mass in older adults: a longitudinal study based on CLHLS

**DOI:** 10.3389/fpsyt.2025.1595625

**Published:** 2025-06-03

**Authors:** Yin Shi, Yu Zhang, Xinyu Yang, Jiali Yang, Shilang Wang, YanFang Hong

**Affiliations:** ^1^ Department of Critical Care Medicine, Deqing People's Hospital, Huzhou, Zhejiang, China; ^2^ College of Medical Science, Huzhou University, Zhejiang, China; ^3^ Department of Nursing, Huzhou Central Hospital, Huzhou, Zhejiang, China; ^4^ Department of Neurosurgery, The Second People’s Hospital of Yuhuan, Taizhou, Zhejiang, China

**Keywords:** older adults, cognitive function, muscle mass, cross lag, restricted cubic splines

## Abstract

**Background:**

Sarcopenia is the main cause of disability in an aging society and increases the risk of death in older adults. However, the relationship between cognitive function and muscle mass and the underlying mechanisms are not clear. This study aims to investigate the relationship between cognitive function and muscle mass in the older adults.

**Methods:**

This study was based on the Chinese Longitudinal Healthy Longevity Survey (CLHLS), phase III from 2011 to 2018. We analyzed 2536 participants aged ≥60 years. SPSS 27.0 software was used for data screening and statistical analysis, and MPLUS 8.7 and R4.4.2 software were used to construct cross-lag models and restricted cubic splints.

**Results:**

In this study, out of 2,536 participants, there were 1,283 males (50.6%) and 1,253 females (49.4%), with an average age of 77.54 ± 8.6 years. Correlation analysis showed that cognitive function was positively correlated with muscle mass in older adults. At all time points (*P*<0.05). The cross-lag model revealed a one-way prediction effect: The path coefficients of ASMI→MMSE in T1→T2 and T2→T3 were statistically significant in the general population, men and women (*P*<0.05), and the path coefficients *β* were all greater than 0. The association of MMSE → ASMI was significant only at the T2 → T3 time point in the overall population (β = 0.010, P < 0.05), and not statistically significant at T1 → T2 and T2 → T3 time points in both males and females (P <0.05). RCS results showed that the association between skeletal muscle mass and cognitive impairment in the total population (*P*_overall trend <_0.05, *P*_non-linear <_0.05), older men (*P*_overall trend <_0.05, *P*_non-linear <_0.05) and older women (*P*_overall trend <_0.05, *P*_non-linear <_0.05) showed a nonlinear increasing trend. It is suggested that ASMI should be maintained at 7.45kg/m^2^ and 5.68kg/m^2^ or above in older men and women, respectively.

**Conclusion:**

Muscle mass had a major predictive effect on cognitive trajectory, especially in females. Maintaining ASMI above gender-specific thresholds may help slow cognitive decline, suggesting that muscle mass can serve as an adjustable biomarker for dementia prevention. Longitudinal studies should verify the validity of these thresholds in different populations.

## 1 Introduction

As a major challenge to human society in the 21st century, population aging has become an irreversible global trend. It is projected that by 2050, the proportion of individuals aged 65 and above in China will reach 27.9% (1), surpassing the United Nations’ threshold of 7%, signifying China’s transition into a super-aging society. Concurrently, cognitive dysfunction, closely associated with the aging process, has emerged as a significant concern. Cognitive impairment not only severely affects core cognitive domains such as judgment and attention in older adults (2) but also poses a risk of progressing to irreversible dementia (3), thereby becoming a critical factor impacting the healthy quality of life for older adults. Research has confirmed a significant positive correlation between cognitive impairment and aging (4).

Additionally, studies have demonstrated that factors such as education level, obesity status, lifestyle, and vascular-related conditions can influence cognitive function differentiation in older adults (5). Notably, cognitive function is a key biomarker for predicting healthy life expectancy in older adults (6). However, current clinical interventions remain limited to symptom management, lacking effective etiological treatments (7). Therefore, identifying and managing risk factors for cognitive impairment at an early stage holds great practical significance and urgency in the field of older adults’ health.

Sarcopenia, a common and progressive skeletal muscle disease characterized by muscle atrophy and reduced strength and quality (8), significantly impairs physical function and increases the risk of falls, fractures, and mortality in older adults (9), profoundly affecting their quality of life. Consequently, it has garnered considerable attention in both academic and clinical fields. According to the latest definition of sarcopenia (10), low muscle mass is a crucial diagnostic criterion. From a physiological perspective, muscle mass plays an essential role in regulating energy metabolism, influencing insulin sensitivity, and maintaining overall bodily function (11).

Given that muscle mass and cognitive function share neuroendocrine regulatory pathways (such as the insulin signaling pathway) and inflammatory mechanisms (11, 12), numerous studies have explored the complex relationship between muscle mass and cognitive function. Recent epidemiological studies have shown that older adults with lower muscle mass are at higher risk of cognitive decline and neurodegenerative diseases like Alzheimer’s (13–15). These findings suggest an intrinsic link between muscle mass and cognitive function. Mechanistic studies indicate that muscle-derived factors, such as myokines, may be released into the bloodstream to influence neuroplasticity, neurotransmitter regulation, and inflammatory responses in the brain, all of which are closely related to cognitive function (16, 17). Animal models and human trials further support this relationship (18–20).

The current research landscape is marred by several significant gaps. Firstly, the majority of Chinese studies are cross-sectional, which constrains the ability to draw causal conclusions (21, 22). Secondly, longitudinal analyses tend to concentrate on one-way relationships, overlooking the possibility of reverse causality (23). Thirdly, despite ample documentation of gender differences in muscle mass and cognitive function, there is a dearth of research that systematically investigates gender-specific thresholds for muscle mass in terms of cognitive protection (24–26). Finally, existing dose-response analyses frequently hinge on linear assumptions (27), potentially missing out on nonlinear dynamics that are crucial for clinical interventions.

This scholarly endeavor seeks to bridge the existing knowledge gaps by utilizing longitudinal data sourced from the Chinese Longitudinal Healthy Longevity Survey (CLHLS). We have adopted a Cross-Lagged Panel Model (CLPM) to scrutinize the reciprocal temporal dynamics between muscular mass and cognitive faculties, further enhanced by a Restricted Cubic Spline (RCS) analysis to delineate thresholds that are specific to each gender. Our research is guided by a dual purpose: firstly, to ascertain the directional precedence between muscular mass and cognitive deterioration, and secondly, to formulate actionable Appendicular Skeletal Muscle Index (ASMI) thresholds for the prevention of dementia, specifically calibrated for the elderly demographic in China. By amalgamating bidirectional causality with nonlinear dose-response methodologies, this investigation sheds new light on the interplay between muscle and cognition, furnishing data-driven objectives for the formulation of public health initiatives.

The implications of our findings are profound. Should muscular mass emerge as a more robust harbinger of cognitive trajectories, interventions aimed at combating sarcopenia—such as resistance training and nutritional interventions—could be accorded precedence in strategies for dementia prevention. Additionally, the identification of gender-specific thresholds may enable a more nuanced approach to personalized care, effectively addressing the disparities in muscular composition and hormonal profiles observed between elderly males and females.

## 2 Methods

### 2.1 Data sources and subjects

**Figure 1:**
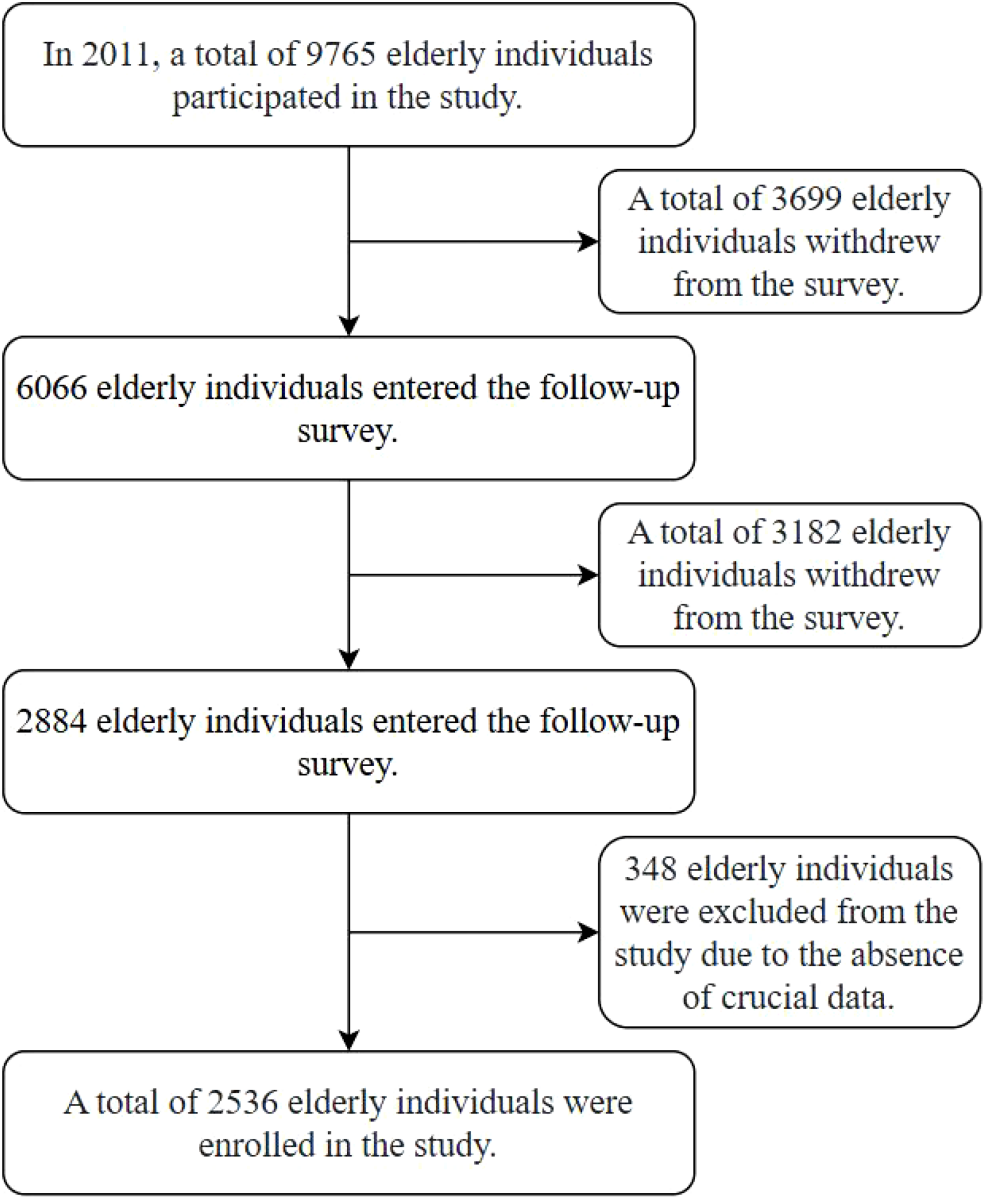
Flowchart of the study population.

The data for this study were sourced from the CLHLS, which encompasses over 500 sites across 23 provinces, municipalities, and autonomous regions in China. The baseline survey was initiated in 1998, with subsequent follow-ups conducted in 2000, 2002, 2005, 2008, 2011, 2014, and 2018. Data usage rights were obtained through the Open Research Data Platform of Peking University. In this study, a total of 9,765 elderly individuals were surveyed in 2011 (T1). Follow-up assessments in 2014 (T2) and 2018 (T3) revealed deaths and dropouts among the elderly participants. After excluding 348 subjects with missing key variable information, a final sample of 2,536 elderly individuals was included in the analysis. See **Figure 1** for details.

### 2.2 Survey tools

#### 2.2.1 Mini-mental state examination

The dataset incorporates the Chinese MMSE scale to evaluate cognitive performance. This 24-question tool covers aspects such as overall capability, responsiveness, concentration, arithmetic skills, memory, linguistic understanding, and self-regulation. Scores are distributed from 0 to 30, with thresholds for cognitive impairment varying by educational background: ≤17 for the non-literate, ≤20 for those with primary education, and ≤24 for individuals with junior high school education or higher. A higher score indicates superior cognitive health (28).

#### 2.2.2 Muscle mass

Appendicular skeletal muscle index (ASMI) (29), adjusted for height, was used to assess muscle mass. Appendicular skeletal muscle mass (ASM) was obtained by using the anthropometric equation for Chinese adults developed by Xu Wen (30) and other scholars. ASM= 0.193 × weight (kg) + 0.107 × height (cm) -4.157 × gender (male =1, female =2) -0.037 × age (years) -2.631. Previous studies have shown (24) that the ASM calculated by this formula is consistent with the results of dual-energy X-ray absorptiometry, and can be used as an economical and feasible index to evaluate the appendicular skeletal muscle mass. After adjusting height, ASMI can be obtained: ASMI=ASM/height (m)^2^.

#### 2.2.3 Covariates

The covariates included in this study included age, place of residence, gender, pension status, multimorbidity, smoking, drinking, sports, education level, marital status, Basic activities of daily living(BADL), Instrumental activity of daily living (IADL), and body mass index (BMI). Multimorbidity was defined as the simultaneous presence of two or more chronic diseases (31). BADL was assessed using a six-item scale developed by Katz (32), which has a total score ranging from 6 to 18 points. Lower scores indicate better BADL. IADL was assessed by the scale developed by Lawton (33), the total score ranged from 8 to 24, the lower the score, the better the IADL. BMI= weight/height^2^ (kg/m^2^) (34).

### 2.3 Statistical methods

Statistical analyses were conducted using SPSS 27.0 and Mplus 8.7 software. Quantitative data were categorized into normally distributed and non-normally distributed variables, with the former described by *x* ± *s*, and the latter by *M(P*_25_ *, P*_75_ *)*. Categorical data were presented as frequencies and percentages. To assess differences in general data between genders, we employed independent sample t-tests, rank sum tests, and chi-square tests. For examining variations in cognitive function and muscle mass among older adults at distinct time points, we utilized repeated measures ANOVA and the Friedman test. Correlations between variables were assessed using Pearson or Spearman correlation coefficients. Cross-lagged regression models were formulated to investigate the temporal causality between cognitive function and muscle mass. To elucidate the dose-response relationship, we applied the restricted cubic spline (RCS) model, selecting the optimal model based on the lowest Akaike information criterion (*AIC*). In conclusion, we explored the relationship between muscle mass and cognitive dysfunction across different subgroups based on the COX model. The significance level was set at *α*=0.05.

## 3 Results

### 3.1 General information

A total of 2536 participants were included and followed up twice for 7 years. There were 1283 males (50.6%) and 1253 females (49.4%), with an average age of (77.54 ± 8.6) years. Independent sample t-test or *x^2^* test showed that MMSE and ASMI differed significantly between males and females (*P*<0.05). See **Table 1** for details.

**Table 1:** General information (*N*=2536).

Characteristics		Total	Male	Female	*P*
Age (years)^a^		77.54 ± 8.6	76.49 ± 8.02	78.62 ± 9.04	<0.05
Place of residence ^b^	City	350 (13.8)	168 (13.09)	182 (14.53)	0.035
	Counties and towns	763 (30.09)	378 (29.46)	385 (30.73)	
	Rural	1423 (56.11)	737 (57.44)	686 (54.75)	
Pension status ^b^	With family	2037 (80.32)	1079 (84.1)	958 (76.46)	<0.05
	Living alone	474 (18.69)	188 (14.65)	286 (22.83)	
	Nursing home	25 (0.99)	16 (1.25)	9 (0.72)	
Multimorbidity^b^	Yes	770 (30.36)	355 (27.67)	415 (33.12)	<0.05
	No	1766 (69.64)	928 (72.33)	838 (66.88)	
Smoking ^b^	Yes	542 (21.37)	480 (37.41)	62 (4.95)	<0.05
	No	1994 (78.63)	803 (62.59)	1191 (95.05)	
Drinking ^b^	Yes	530 (20.9)	427 (33.28)	103 (8.22)	<0.05
	No	2006 (79.1)	856 (66.72)	1150 (91.78)	
Exercise ^b^	Yes	1009 (39.79)	522 (40.69)	487 (38.87)	0.349
	No	1527 (60.21)	761 (59.31)	766 (61.13)	
Education ^b^	Illiteracy	1185 (46.73)	352 (27.44)	833 (66.48)	<0.05
	Elementary school	963 (37.97)	628 (48.95)	335 (26.74)	
	Junior high	348 (13.72)	270 (21.04)	78 (6.23)	
	Secondary school and high school	20 (0.79)	14 (1.09)	6 (0.48)	
	College or above	20 (0.79)	19 (1.48)	1 (0.08)	
Marriage ^b^	Married cohabiting	1425 (56.19)	903 (70.38)	522 (41.66)	<0.05
	Married and separated	63 (2.48)	35 (2.73)	28 (2.23)	
	Divorce	6 (0.24)	5 (0.39)	1 (0.08)	
	Widowed	1008 (39.75)	307 (23.93)	701 (55.95)	
	Unmarried	34 (1.34)	33 (2.57)	1 (0.08)	
IADL ^c^		24 (23, 24)	24 (24, 24)	24 (22, 24)	<0.05
BADL ^c^		18 (18, 18)	18 (18, 18)	18 (18, 18)	<0.05
BMI (kg/m^2^ )^a^		23.75 ± 4.28	23.75 ± 4.28	24.18 ± 4.69	<0.05
MMSE (T1)^a^		29 (28, 30)	28.37 ± 2.43	28.01 ± 2.77	<0.05
ASMI (kg/m^2^ ) (T1) ^a^		6.8 ± 1.36	6.8 ± 1.36	6.06 ± 1.42	<0.05

In the table, data marked with superscript 'a' is represented as mean ± standard deviation, data marked with superscript 'b' is presented in the form of n (%), and data marked with superscript 'c' is displayed as *M* (*P*_25_, *P*_75_); ADL, Activity of Daily Living (Instrumental); BADL, Basic Activities of Daily Living; BMI, Body Mass Index; MMSE (T1), Mini-Mental State Examination (baseline score in 2011); ASMI (T1), Appendicular Skeletal Muscle Mass Index (baseline value in 2011).

### 3.2 Correlation analysis of cognitive function and activities of daily living in older adults

**Table 2** shows the status quo of the three measurement time points of MMSE and AMSI of older adults, and the differences in the three scores of the two were statistically significant. Correlation analysis showed that MMSE and AMSI were significantly positively correlated at the same time point (0.289 ≤ *r* ≤ 0.375, *P<*0.001), and also at different time points (0.281 ≤ *r* ≤ 0.369, *P*<0.05). See **Table 3** for details.

**Table 2:** Analysis of differences in cognitive function and muscle mass in older adults at different time points (*N*=2536).

Items	Projects	T1	T2	T3	*P*
The total	MMSE	29 (28, 30)	29 (28, 30)^①^	29( 27, 30)^①②^	< 0.05
	ASMI	6.80 ± 1.36	6.79 ± 1.35^①^	6.78 ± 1.42^①②^	< 0.05
male	MMSE	30 (28, 30)	29 (28, 30)^①^	29(27, 30)^①②^	< 0.05
	ASMI	7.53 ± 0.80	7.52 ± 0.79^①^	7.59 ± 0.86^①②^	< 0.05
female	MMSE	29(27, 30)	29( 27, 30)^①^	29 (25, 30)^①②^	< 0.05
	ASMI	6.06 ± 1.42	6.06 ± 1.40^①^	5.95 ± 1.40^①②^	< 0.05

Compared with T1,^①^ *P<0.*05; Compared with T2, ^②^ *P*<0.05; MMSE, Mini-Mental State Examination; ASMI, Appendicular Skeletal Muscle Mass Index. The MMSE is presented using the *M*(*P*_25_, *P*_75_), while ASMI is displayed with the mean ± standard deviation.

**Table 3:** Correlation analysis of MMSE and AMSI at the same and different time points in older adults (N=2536).

	MMSE(T1)	MMSE(T2)	MMSE(T3)	ASMI(T1)	ASMI(T2)	ASMI(T3)
MMSE(T1)	1					
MMSE(T2)	0.320**	1				
MMSE(T3)	0.324**	0.379*	1			
ASMI(T1)	0.312**	0.289**	0.369**	1		
ASMI(T2)	0.312**	0.289**	0.367**	0.999**	1	
ASMI(T3)	0.309**	0.281**	0.375**	0.916**	0.916**	1

*P<0.05, **P<0.01; MMSE, Mini-Mental State Examination; ASMI, Appendicular Skeletal Muscle Mass Index. MMSE(T1), MMSE(T2), MMSE(T3): Mini-Mental State Examination scores at Time Points 1 (2011), 2 (2014), and 3 (2018), respectively. ASMI(T1), ASMI(T2), ASMI(T3): Appendicular Skeletal Muscle Mass Index measurements at Time Points 1 (2011), 2 (2014), and 3 (2018), respectively.

### 3.3 CLPM analysis of cognitive function and muscle mass in older adults

**Figure 2:**
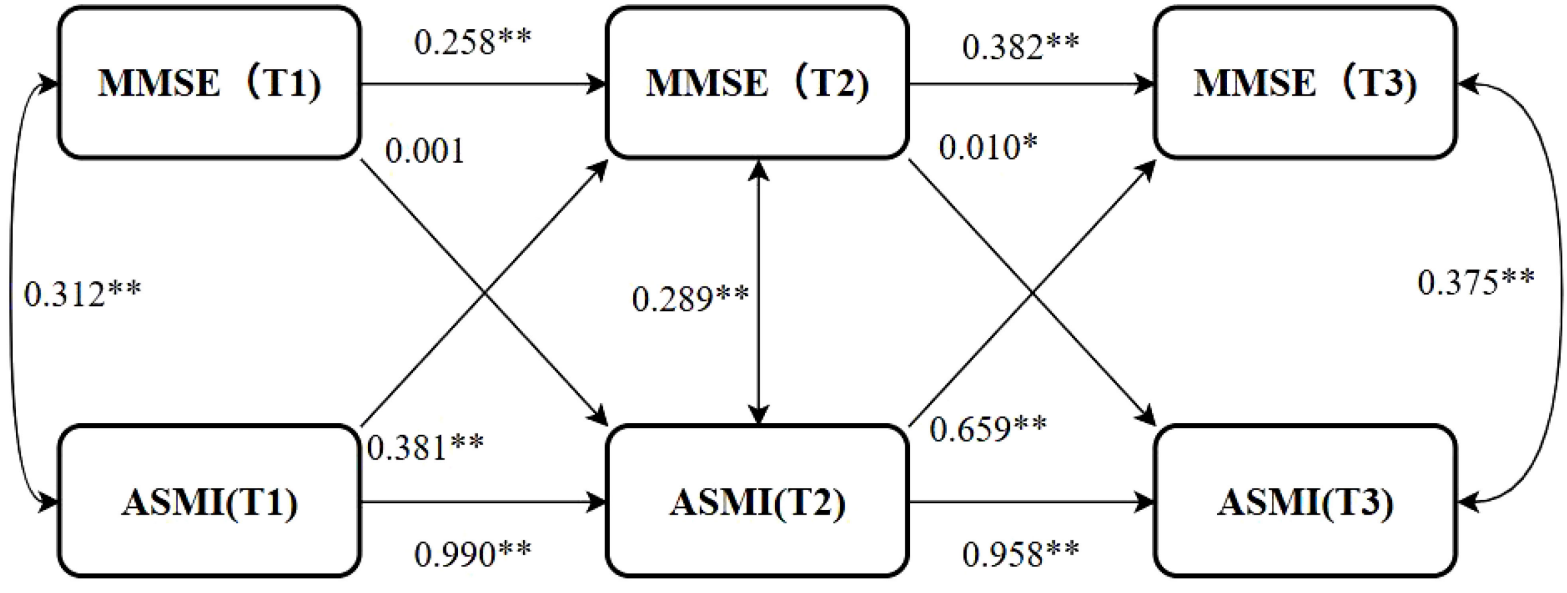
Cross-lag model. *P<0.05, **P<0.01; MMSE(T1), MMSE(T2), MMSE(T3): Mini-Mental State Examination scores at Time Points 1 (2011), 2 (2014), and 3 (2018), respectively. ASMI(T1), ASMI(T2), ASMI(T3): Appendicular Skeletal Muscle Mass Index measurements at Time Points 1 (2011), 2 (2014), and 3 (2018), respectively.

The Cross-lag model was used to analyze the dynamic association between cognitive function and muscle mass, as well as the direction of causality. After controlling the confounding factors such as age, gender, pension, multimorbidity, smoking, drinking, education, marriage, BADL, IADL, and BMI, the model fitted well (*CFI*=0.996>0.90, *TLI*=0.985>0.90, *SRMR=*0.027<0.05, *RMSEA*=0.076<0.08). The autoregression effect suggested that cognitive function and muscle mass at the Tn moment were affected by their own Tn-1 level, suggesting that the stability of cognitive function was at a medium-low level, and the stability of muscle mass was at a high level. The path coefficients of MMSE to ASMI from T1 to T2 were not statistically significant (*P*>0.05*), wh*ile the path coefficients from T2 to T3 were statistically significant (*P*<0.05) *and th*e path coefficient *β*>0, sugges*t*ing that when MMSE decreased to a certain extent, the decline of cognitive function could cause the decline of muscle mass. The path coefficients of ASMI→MMSE in T1→T2 and T2→T3 were statistically significant (*P*<0.05), and the path coefficients *β* were all greater than 0, suggesting that the decline of muscle mass can cause the decline of cognitive function. The path coefficient of the ASMI→MMSE path direction was significantly greater than that of the MMSE→ ASMI path direction, suggesting that the influence of muscle mass on cognitive function was greater than that of cognitive function on muscle mass. The model results are shown in **Figure 2**, and the path coefficients are shown in **Table 4**.

**Table 4:** Path coefficients (*N*=2536).

The path	The total		male		female	
	T1→T2	T2→T3	T1→T2	T2→T3	T1→T2	T2→T3
MMSE autoregressive	0.258**	0.382**	0.205**	0.341**	0.288**	0.407**
ASMI autoregressive	0.990**	0.958**	0.983**	0.852**	0.986**	0.907**
MMSE→ASMI	0.001	0.010*	-0.001	0.008	0.001	0.009
ASMI→MMSE	0.381**	0.659**	0.325**	0.555**	0.351**	0.792**

*P<0.05, **P<0.01; The MMSE is presented using the *M*(*P*_25_, *P*_75_), while ASMI is displayed with the mean ± standard deviation.

When stratified by gender, the other covariates were the same as those in the general population, and the model fitted well across different genders (Male: *CFI* = 0.987, *TLI*=0.955, *SRMR=*0.046, *RMSEA*<0.001; Female: *CFI*=0.998, *TLI*=0.992, *SRMR=*0.018*, RMSEA*=0.042). The results showed that there were gender differences in the causal time sequence relationship between cognitive function and muscle mass in older adults. The autoregressive effect showed that the stability of cognitive function and muscle mass in females was higher than that in males. The path coefficients of ASMI→MMSE in each time period were statistically significant in both men and women (*P<*0.05), and the path coefficients in women were significantly higher than those in men, suggesting that the effect of muscle mass on cognitive function in women was more significant than that in men. However, in the path direction of MMSE→ASMI, there were no significant differences in the path coefficients for males and females from T1→T2 and from T2→T3 (*P*>0.05), indicating that cognitive function does not predict muscle mass in stratified specific analyses. The path coefficients are shown in **Table 4**.

### 3.4 Dose-response relationship between muscle mass and cognitive impairment

**Figure 3:**
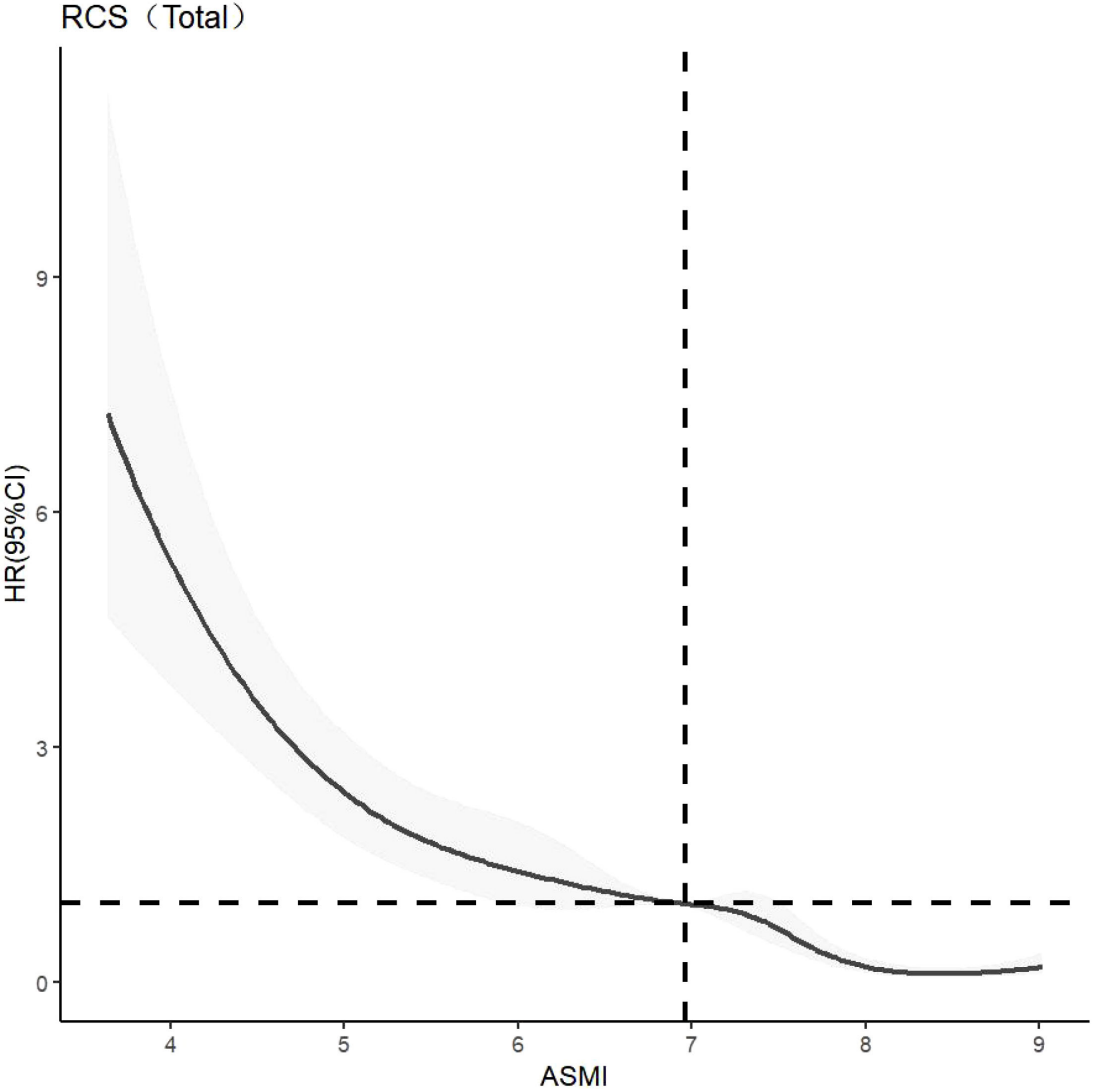
Dose-response relationship between skeletal muscle mass and cognitive impairment in the overall older adults population. RCS, Restricted Cubic Spline Model; HR(95%CI), Hazard Ratio (95% Confidence Interval); ASMI, Appendicular Skeletal Muscle Mass Index.

**Figure 4:**
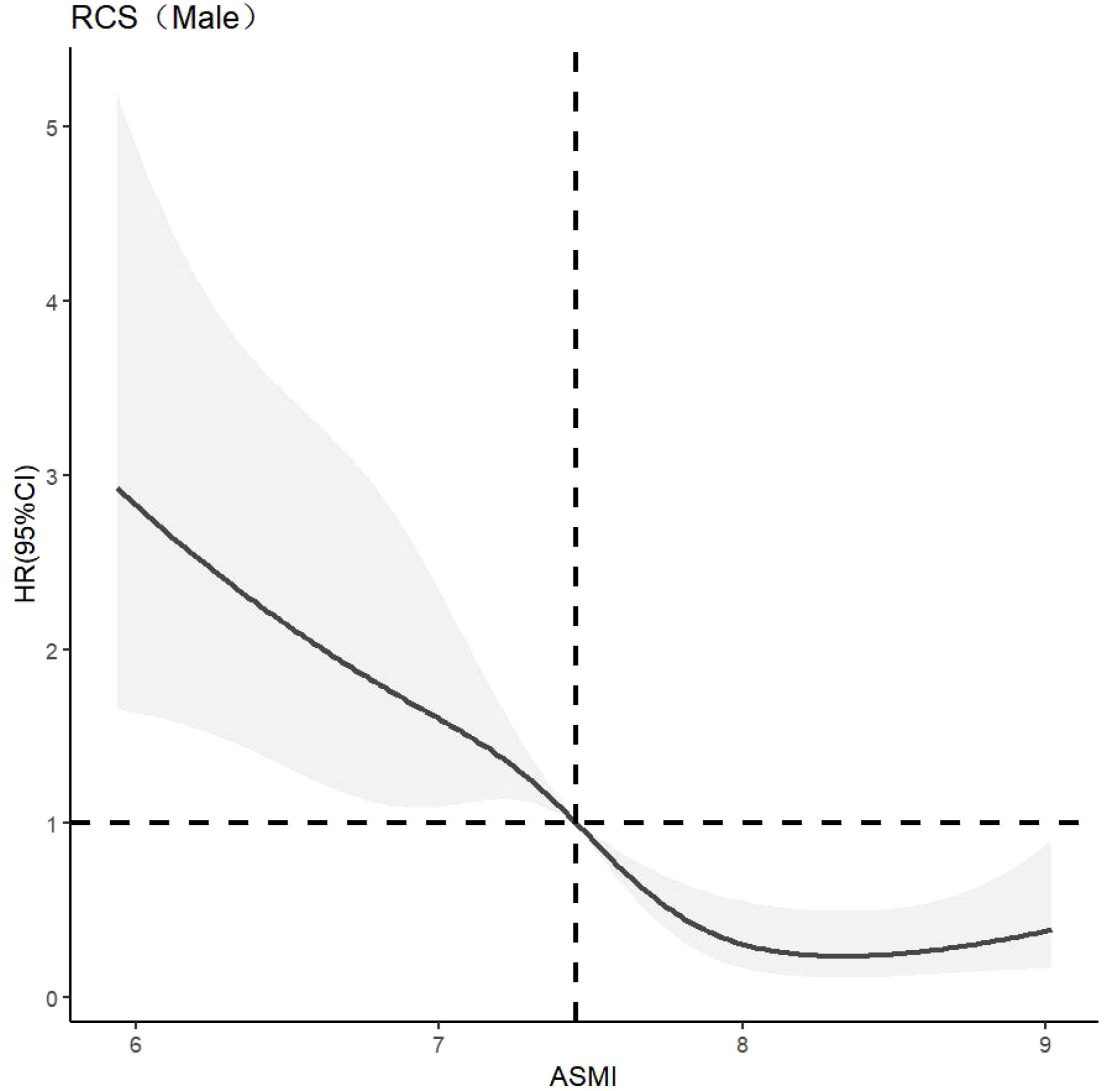
Dose-response relationship between skeletal muscle mass and cognitive dysfunction in male. See **Figure 3** for details.

**Figure 5:**
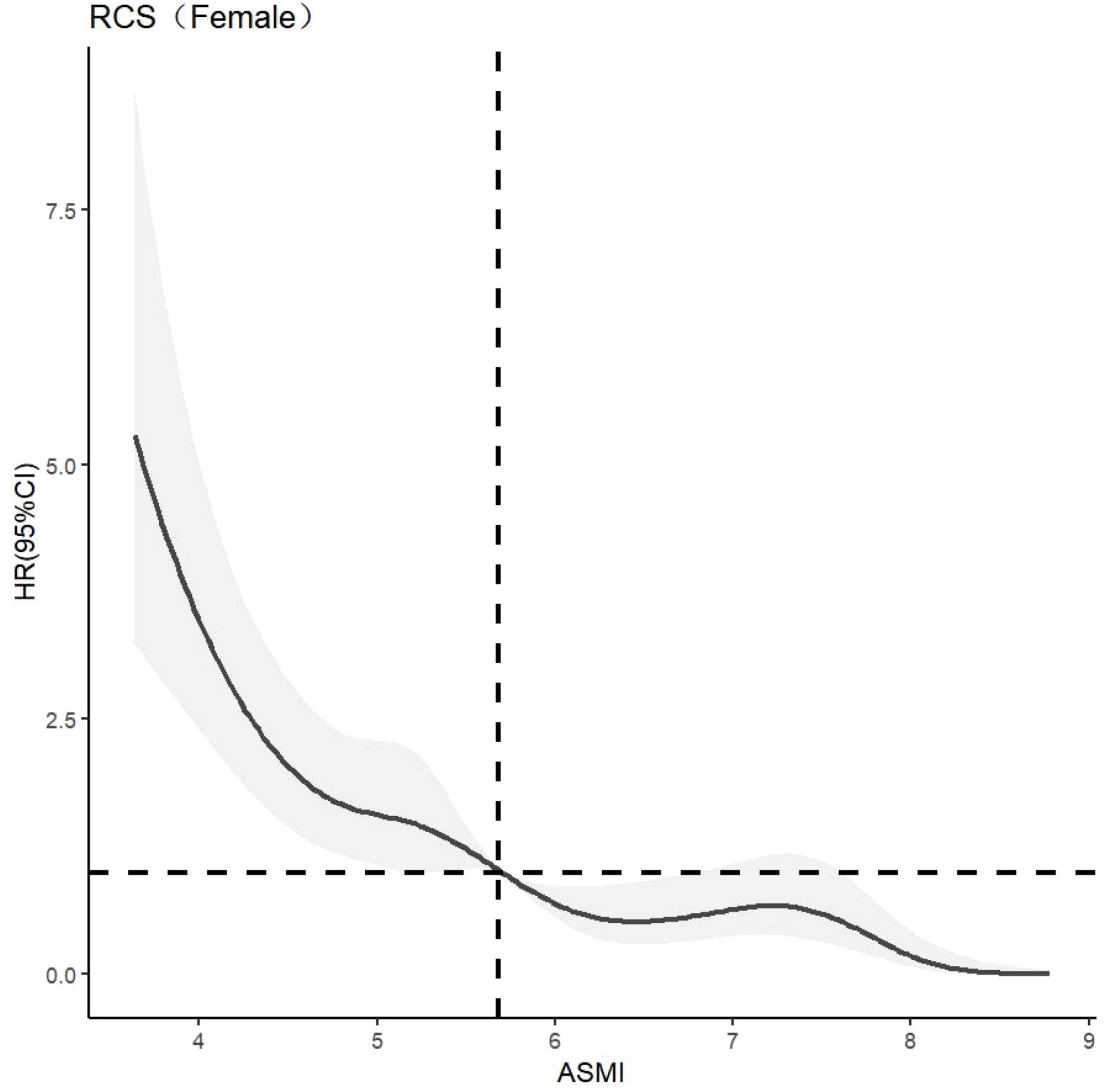
Dose-response relationship between skeletal muscle mass and cognitive dysfunction in female. See **Figure 3** for details.

CLPM analysis showed that the effect of muscle mass on cognitive function was greater than that of cognitive function on muscle mass, highlighting the importance of muscle mass in this relationship. The dose-response relationship between muscle mass and cognitive function was further explored. The optimal number of nodes in this model is 3-7. According to the spline regression coefficient and *AIC* criterion, this study found that in the model related to cognitive dysfunction*, AIC*_total_=6456.58*, AIC*_male_=1908.71, *AIC*_female_=3994.858 reached the minimum, and the number of nodes was 7, 5, and 7 respectively. After controlling for confounding factors such as age, gender, elderly care, smoking, drinking, education, marriage, BADL, IADL, and BMI, there was a nonlinear dose-response relationship between muscle mass and cognitive impairment (*P*_overall trend <_0.05, *P*_non-linear <_0.05). When ASMI < 6.96 kg/m^2^, *HR* > 1, it is suggested that muscle mass is a risk factor for cognitive dysfunction in older adults. After stratified analysis, there was a nonlinear dose-response relationship between muscle mass and cognitive dysfunction in older men (*P*_overall trend <_0.05, *P*_non-linear <_0.05). When ASMI*<*7.45kg/m^2^ *, HR>1*, suggests that muscle mass is a risk factor for cognitive dysfunction in older men at this time. There was a nonlinear dose-response relationship between muscle mass and cognitive dysfunction in older women (*P*_overall trend <_0.05, *P*_non-linear <_0.05). When ASMI <5.68kg/m^2^, *HR >*1, indic*a*ting that muscle mass was a risk factor for cognitive dysfunction in older women. The model results are shown in **Figures 3**–**5**.

### 3.5 Subgroup analysis

**Figure 6:**
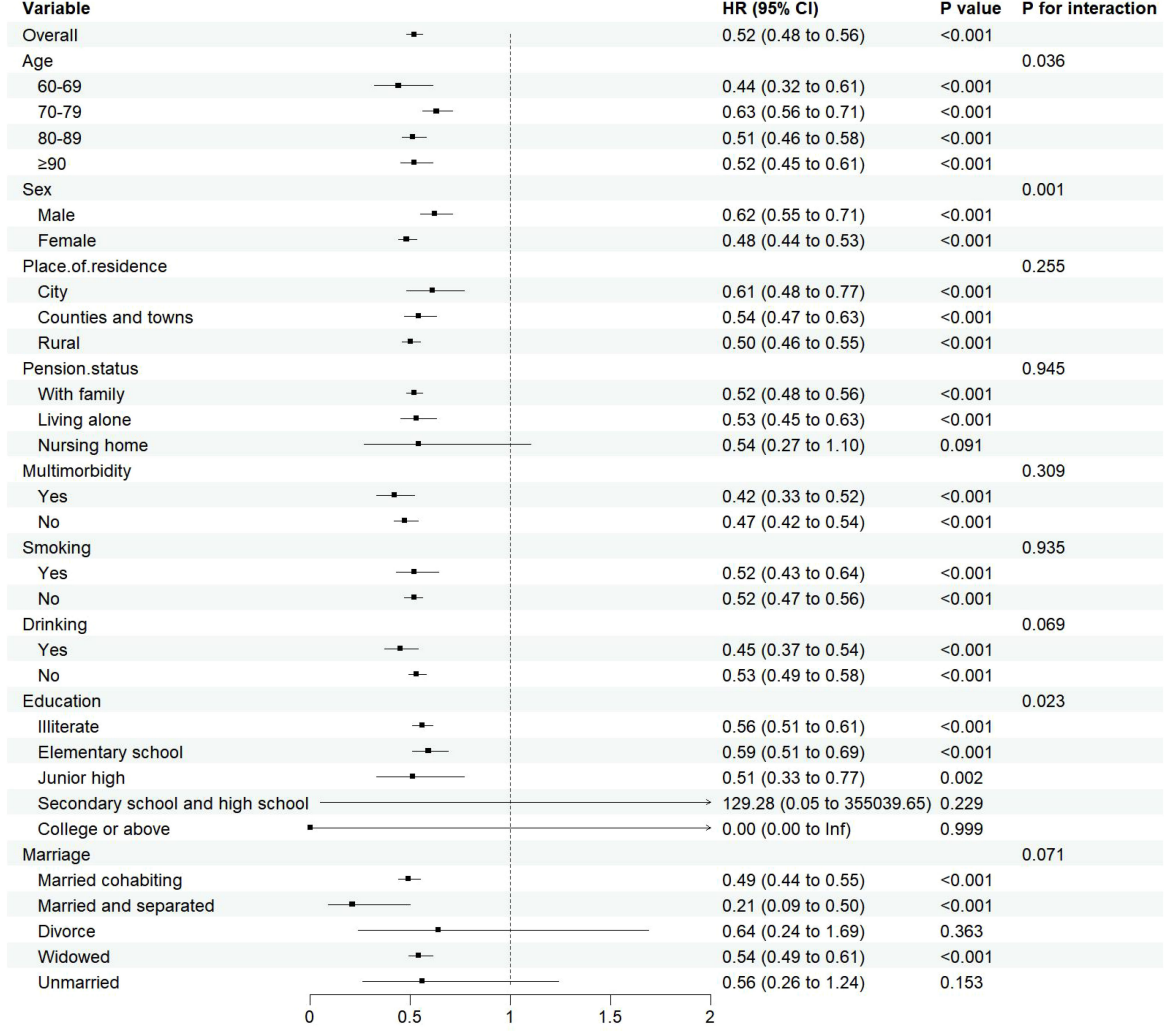
Subgroup analysis results.

As age advances, the relationship between sarcopenia and cognitive impairment in the elderly becomes increasingly evident, prompting this study to perform age-stratified subgroup analyses. This methodological choice bolsters the research’s credibility and persuasiveness while mitigating the risk of biases. The subgroup analyses, employing the COX regression model, demonstrated a correlation between decreased muscle mass and the onset of cognitive dysfunction in specific elderly subgroups. Interaction tests further exposed notable variations in the link between muscle mass and cognitive function across different age, gender and education(*P*_for interaction <_0.05). Notably, the 60-69 age cohort exhibited the most robust association (*HR*=0.44, 95%*CI*:0.32~0.61), with females exhibiting a more pronounced protective effect (*HR*=0.48, 95%*CI*:0.44~0.53) than males (*HR*=0.62, 95%*CI*:0.55~0.71). Additionally, individuals with lower levels of education displayed a consistent protective effect (*HR*=0.51-0.59, all *P*<0.05.), suggesting that the influence of muscle mass on cognitive health is multifaceted and influenced by a range of demographic factors.The detailed results can be found in **Figure 6**.

## 4 Discussion

The present study, conducted as a longitudinal investigation, revealed a bidirectional relationship between muscle mass and cognitive function. Specifically, muscle mass can predict changes in cognitive function, while cognitive dysfunction at certain stages can reciprocally predict changes in muscle mass. Moreover, the influence of muscle mass on cognitive function appears to be more pronounced, particularly evident in women.

The results suggest that cognitive function and muscle mass are interpredictive in older adults. Previous systematic reviews and meta-analyses, such as those by Gao Qianqian, have identified cognitive impairment as a risk factor for sarcopenia (22). This study refines this causal inference through its longitudinal design, observing for the first time the predictive role of cognitive function on muscle mass in early stages. Cognitive decline in older adults is associated with reduced orientation and memory. When this decline reaches a critical level, it leads to diminished daily living abilities, decreased physical activity, and inadequate nutritional intake, ultimately resulting in reduced muscle mass (22). Consistent with our findings, Beeri reported in a U.S.-based longitudinal study that muscle mass loss predicted cognitive impairment (23). This may be due to decreased muscle mass increasing the incidence of adverse clinical outcomes like falls, fractures, and physical disability, which limit activity and reduce social participation, further exacerbating cognitive impairment (35).

Additionally, our study found that the impact of muscle mass on cognitive function was more significant than the reverse effect. This may be because muscle activity directly influences basic physiological processes of brain health and function. Muscle activity stimulates the production of nerve growth factors, improves blood circulation, increases oxygen and nutrient supply to the brain, and affects hormone levels such as testosterone and growth hormone, thereby promoting brain health and neuroplasticity (13, 36, 37), which in turn influences cognitive function.

General information indicates that men typically exhibit higher levels of cognitive function than women, consistent with Sivaniya’s study (24). Some scholars (25) consider the female gender a risk factor for cognitive dysfunction, possibly due to post-menopausal reductions in estrogen and related hormones, as well as differences in brain structure, leading to lower cognitive function in women compared to men. Similarly, muscle mass is generally higher in men than in women, reflecting gender-based differences in body composition. Women typically have a higher body fat percentage and lower muscle mass than men (26). Additionally, older women have longer life expectancies but may experience lower cognitive function and muscle mass due to the cumulative effects of less physical exercise, poorer health status, and broader differences in social and family roles (38).

Due to these gender differences, stratified analysis revealed that cognitive function was more significantly affected by muscle mass in women than in men, consistent with Lee (39). These differences are supported by gender-specific physiological factors, where gender hormones influence sarcopenia and hippocampal plasticity, activation, and morphology. Estradiol not only regulates muscle satellite cell proliferation (e.g., by promoting MyoD expression) through ERα receptors (40) but also enhances synaptic plasticity in the central nervous system via the BDNF-TrkB pathway (41), which is more active in females, playing a key role in gender differences.

Further analysis identified a nonlinear dose-response relationship between muscle mass and the risk of cognitive impairment. As muscle mass decreases, the risk of cognitive impairment increases nonlinearly. The study suggests maintaining ASMI above 6.96 kg/m² in older adults, 7.45 kg/m² in older men, and 5.68 kg/m² in older women. This aligns with the Asian Consensus for the Diagnosis of Sarcopenia (7.0 kg/m² for men and 5.4 kg/m² for women) (22), indicating that these values can serve as targets for cognitive protective interventions. Demura’s study (27) also found similar results but used only linear and logistic regression, which did not fully capture the dynamic change trajectory of muscle mass and its impact on cognitive function. The recommended value of ASMI is higher in men than in women, which may be related to the difference in body composition between men and women. Fat mass is generally higher in women than in men, and the initial muscle mass is also higher in men than in women (26). This finding is consistent with the gender differences that Xu Wen (30) considered when developing the anthropometric equation.

Subgroup analysis in this study has uncovered the heterogeneity in the protective association between muscle mass and cognitive function across different groups, suggesting that the underlying mechanisms may involve the interplay of biological and social factors. Age-stratified analysis reveals that the strongest association is found in the 60-69 age group, possibly linked to a critical biological window during the transition from middle to old age: at this stage, mitochondrial function in skeletal muscle has not yet significantly declined (42), and neurotrophic factors secreted by muscles remain at high levels (43), both of which synergistically enhance neural plasticity. After the age of 70, the association weakens, potentially due to the exacerbation of aging-related systemic inflammation and insulin resistance, pathological processes that can disrupt the muscle-brain metabolic coupling (44). Gender differences show that women exhibit a stronger protective effect, which has been previously discussed. Regarding education level, the protective effect is stable in those with low education, but in the highly educated subgroup, the sample size is severely insufficient (only 0.79%), leading to statistical overfitting in the Cox proportional hazards model and rendering these results statistically insignificant. This insufficiency in sample size may lead to extreme hazard ratios that do not accurately reflect the actual situation.

The strength of this study is that the cross-lagged regression model based on a nationally representative sample of the CLHLS database was used for a longitudinal study to continuously observe the changes in muscle mass and cognitive function, thereby more precisely revealing the causal temporal relationship between them. In addition, this study further explored the dose-response relationship between muscle mass and cognitive function to avoid the limitations caused by categorical data and make the effect of muscle mass on cognitive function clearer. This study also has limitations. Firstly, although a standardized scale was used to assess cognitive function, it may still be affected by environmental interference and recall bias. Secondly, the use of relative skeletal muscle mass index based on weight, height, weight, and age may be insufficient to assess muscle mass, such as difficulty in taking into account individual differences. Therefore, it is necessary to further explore the related indicators that affect muscle mass in the future to fully reveal the biological mechanism and dynamic relationship between muscle mass and cognitive function in older adults.

## 5 Conclusion

Based on the analysis of longitudinal data from the CLHLS cohort, this study revealed a dynamic bidirectional association between muscle mass (ASMI) and cognitive function (MMSE) in older adults, with significant gender heterogeneity. The results of this study suggest that improving muscle mass through reasonable dietary intervention and increasing physical exercise may have a positive effect on cognitive function in older adults, which provides a relevant reference for cognitive intervention strategies in older adults.

## Data Availability

The datasets presented in this study can be found in online repositories. The names of the repository/repositories and accession number(s) can be found below: https://opendata.pku.edu.cn/dataverse/CHADS.
